# Habit acquisition in the context of neuronal genomic and epigenomic mosaicism

**DOI:** 10.3389/fnhum.2014.00255

**Published:** 2014-04-25

**Authors:** Francisco J. Novo

**Affiliations:** Biochemistry and Genetics, University of NavarraPamplona, Spain

**Keywords:** habits, epigenomics, mosaicism, metaplasticity, genomics

A recent paper (Gräff et al., 2014) shows that remote fear memories in mice can be stably attenuated with the administration of histone de-acetylase (HDAC) inhibitors during reconsolidation. This achieved persistent attenuation of remote memories, even though it is well established that the brief period of hippocampal neuroplasticity induced by recent memory recall is absent for remote memories. Apparently, such epigenetic intervention primed the expression of neuroplasticity-related genes.

This work comes shortly after the finding (McConnell et al., 2013) that individual neurons show an extraordinary degree of genomic mosaicism. Sequencing the genomes of single human frontal cortex neurons, these authors found that up to 41% of neurons contain at least one *de novo* copy-number variant (CNV) of at least one megabase in size. Segmental duplications have greatly expanded in African great apes (Marques-Bonet et al., 2009), and it is possible that increased retrotransposon activity during human neurogenesis also contributes to this striking diversity in CNV numbers in neuronal genomes (Singer et al., 2010).

Taken together, both studies support the notion that genomic and epigenomic mosaicism allows for the introduction of heritable changes at the single-cell level that promote neuronal plasticity, and thus help to explain how human actions can modify neural circuits involved in memory and learning.

## (Epi) genomic mosaicism and synaptic plasticity

The epigenomic basis of memory and learning is an active field of research in neuroscience (Mehler, 2008; Baker-Andresen et al., 2013). Long-term memory (LTM) formation requires the consolidation of short-term memories, so that these can be later recalled to participate in a wide range of behavioral responses such as making decisions based on previous knowledge (Puckett and Lubin, 2011). Studies about chromatin modifications in various brain regions have shown that learning experiences can trigger epigenetic changes that mediate synaptic long-term potentiation and contribute to LTM consolidation (Guo et al., 2011).

DNA methylation is a well-studied type of epigenetic modification. Cortical DNA methylation is one of the molecular mechanisms used by the brain to preserve remote memories (Miller et al., 2010) and regulates associative reward learning (Day et al., 2013). Changes in DNA methylation at specific genomic sites can modulate the expression of genes involved in synaptic plasticity and memory suppression, thus leading to memory consolidation. For example, knockout mice for methyltransferases DNMT1 or DNMT3A that lose DNMT activity in the hippocampus are unable to form new memories, indicating the importance of dynamic DNA methylation in the process of LTM formation (Feng et al., 2010). However, it is interesting that a number of CpGs differentially methylated in response to neuronal activity might not lead to stable changes in transcription, but rather prime the genome to respond to future stimuli. In the context of memory processing, experience-mediated variations in DNA methylation represent a type of genomic metaplasticity that could prime the transcriptional response and facilitate neuronal reactivation (Baker-Andresen et al., 2013).

In addition to DNA methylation, other epigenetic marks such as histone methylation and acetylation have been shown to play crucial roles in memory and learning processes (Mehler, 2008). For instance, certain histone methylation marks such as the tri-methylation of lysine 4 in histone 3 (H3K4me3) and the di-methylation of lysine 9 (H3K9me2), activate and repress gene transcription, respectively, in the hippocampus during fear–memory consolidation (Gupta et al., 2010).

In summary, experience-driven changes in various epigenetic marks could direct neuronal plasticity in several ways: regulating alternative splicing of specific genes, releasing transposable elements from transcriptional silencing, or creating bivalent chromatin domains that render genes poised for transcription (Baker-Andresen et al., 2013). Reactivation of transposable elements might be particularly relevant in the context of neuronal mosaicism, as it has been shown that L1 retrotransposons are transiently released from epigenetic suppression during neurogenesis so they can mobilize to different loci in individual cells. This would lead to genomic rearrangements that might enable different neurobiological processes, including neural plasticity (Singer et al., 2010; Baillie et al., 2011).

## Habits and (epi) genomic mosaicism

The genomic basis of neuronal plasticity and metaplasticity is particularly relevant in the context of human habits. From a neuroscientific perspective, habits arise from the repeated learning of associations between actions and their contextual features. In this regard, a fundamental issue in neuroscience will be the relationship between habit acquisition and neuronal (epi) genomic mosaicism in humans.

Recent advances in single-cell genomics and non-invasive imaging technologies suggest that significant developments will be achieved in the near future. Once neuronal circuits involved in habit learning are identified by imaging studies, the analysis of genomic and epigenomic neuronal mosaicism should reveal which changes facilitate (or result from) habit acquisition. This will require the development of techniques for the analysis of genomes and epigenomes in single-cells, and imaging technologies that capture epigenetic changes *in vivo*.

In this regard, single-cell genome sequencing is shedding new light into the genetic architecture and variability between cells, highlighting the dynamic nature of the genome (Blainey and Quake, 2014). Although single-cell epigenomics is still in its infancy, the use of a microfluidic platform has recently boosted efficiency and allowed the analysis of DNA methylation in six genes simultaneously in one cell (Lorthongpanich et al., 2013). Such advances will help to read the epigenomes of individual neurons obtained from brain surgery or post-mortem samples.

At the same time, new molecular imaging strategies are being implemented to monitor microRNA biogenesis and its post-transcriptional regulation, *in vivo* as well as *in vitro*, using several reporter systems such as fluorescent proteins, bioluminescent enzymes, molecular beacons, and/or various nanoparticles (Hernandez et al., 2013). For instance, an *in vivo* luciferase imaging system was used to monitor miR-221 biogenesis (Oh et al., 2013). Although non-invasive analysis of gene expression is still in the initial stages of development, molecular imaging of genomic and epigenomic changes might become a reality in a not-so-distant future. Then, it will be possible to design experiments to investigate how genomic and epigenomic mosaicism facilitate (or are influenced by) the acquisition of habits.

### Conflict of interest statement

The author declares that the research was conducted in the absence of any commercial or financial relationships that could be construed as a potential conflict of interest.
